# Crystal structure of ethyl 5-[3-(di­methyl­amino)­acrylo­yl]-2-{[(di­methyl­amino)­methyl­idene]­amino}-4-methylthio­phene-3-carb­oxy­late

**DOI:** 10.1107/S2056989015018885

**Published:** 2015-11-04

**Authors:** M. S. Krishnamurthy, N. L. Prasad, H. Nagarajaiah, Noor Shahina Begum

**Affiliations:** a Department of Studies in Chemistry, Central College Campus, Bangalore University, Bangalore 560 001, Karnataka, India

**Keywords:** crystal structure, thio­phene derivative, hydrogen bonding, C—H⋯π inter­action

## Abstract

In the title compound, C_16_H_23_N_3_O_3_S, the dihedral angles between the thio­phene ring and the almost planar di­methyl­amino-methyl­ene­amino (r.m.s. deviation = 0.005 Å) and di­methyl­amino-acryloyl (r.m.s. deviation = 0.033 Å) substituents are 6.99 (8) and 6.69 (7)°, respectively. The ester CO_2_ group subtends a dihedral angle of 44.92 (18)° with the thio­phene ring. An intra­molecular C—H⋯O hydrogen bond generates an *S*(6) ring. In the crystal, inversion dimers linked by pairs of C—H⋯O hydrogen bonds generate *R* ^2^_2_(14) loops. In addition, a weak C—H⋯π inter­action is observed.

## Related literature   

For the biological activitivity of thio­phene derivatives, see: Rizwan *et al.* (2014 ▸); Mishra *et al.* (2011 ▸); Sabnis *et al.* (1999 ▸). Mabkhot *et al.* (2013 ▸). For synthetic background, see: Gewald *et al.* (1966 ▸).
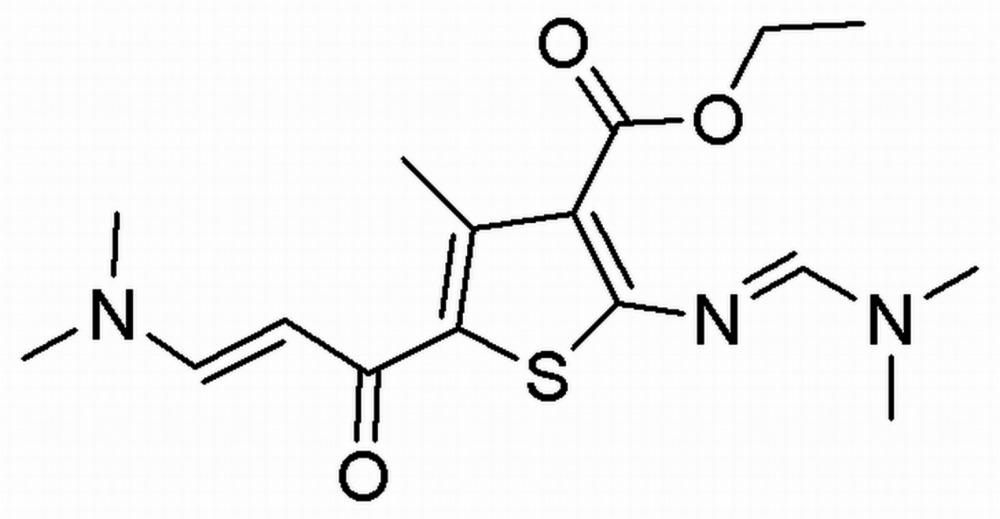


## Experimental   

### Crystal data  

C_16_H_23_N_3_O_3_S
*M* *_r_* = 337.43
Triclinic, 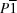

*a* = 7.6954 (5) Å
*b* = 8.1799 (5) Å
*c* = 13.9626 (9) Å
α = 95.928 (2)°
β = 103.685 (2)°
γ = 90.137 (2)°
*V* = 849.07 (9) Å^3^
*Z* = 2
Mo *K*α radiation
μ = 0.21 mm^−1^
*T* = 100 K
0.17 × 0.16 × 0.15 mm

### Data collection  

Bruker SMART APEX CCD diffractometer
Absorption correction: multi-scan (*SADABS*; Bruker, 1998 ▸) *T*_min_ = 0.963, *T*_max_ = 0.967
5876 measured reflections
2991 independent reflections
2646 reflections with *I* > 2σ(*I*)
*R*_int_ = 0.016

### Refinement  

*R*[*F* ^2^ > 2σ(*F* ^2^)] = 0.032
*wR*(*F* ^2^) = 0.089
*S* = 1.02
2991 reflections
214 parameters
H-atom parameters constrained
Δρ_max_ = 0.27 e Å^−3^
Δρ_min_ = −0.19 e Å^−3^

### 

Data collection: *SMART* (Bruker, 1998 ▸); cell refinement: *SAINT-Plus* (Bruker, 1998 ▸); data reduction: *SAINT-Plus*; program(s) used to solve structure: *SHELXS97* (Sheldrick, 2008 ▸); program(s) used to refine structure: *SHELXL97* (Sheldrick, 2008 ▸); molecular graphics: *ORTEP-3 for Windows* (Farrugia, 2012 ▸) and *CAMERON* (Watkin *et al.*, 1996 ▸); software used to prepare material for publication: *WinGX* (Farrugia, 2012 ▸).

## Supplementary Material

Crystal structure: contains datablock(s) global, I. DOI: 10.1107/S2056989015018885/hb7489sup1.cif

Structure factors: contains datablock(s) I. DOI: 10.1107/S2056989015018885/hb7489Isup2.hkl

Supporting information file. DOI: 10.1107/S2056989015018885/hb7489Isup3.cml

**Fig. 1.**
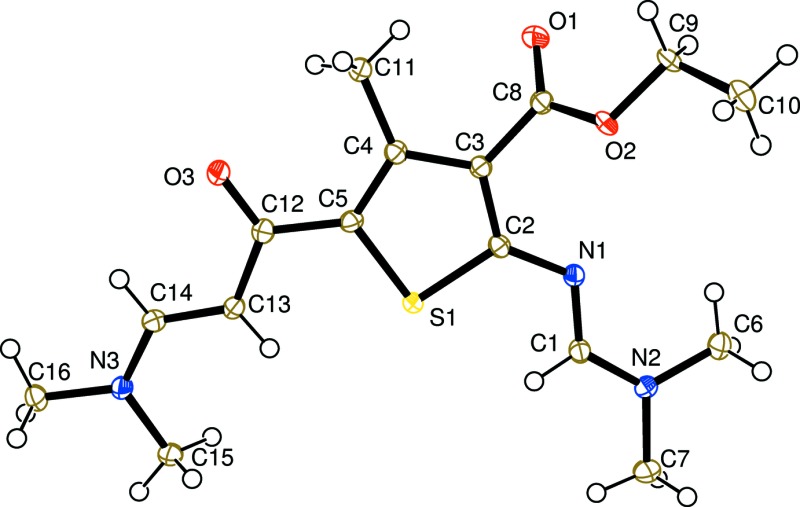
The molecular structure of the title compound with displacement ellipsoids drawn at the 50% probability level.

. DOI: 10.1107/S2056989015018885/hb7489fig1.tif
The mol­ecular structure of the title compound with displacement ellipsoids drawn at the 50% probability level.

**Fig. 2.**
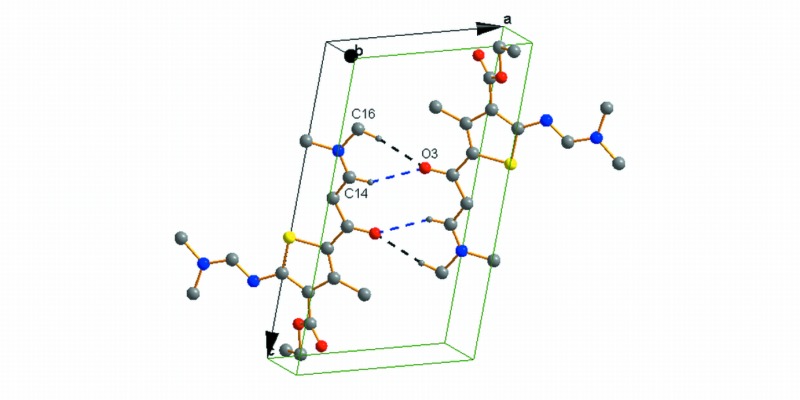
Unit cell packing of the title compound showing intermolecular C—H···O interactions with dotted lines. H-atoms not involved in hydrogen bonding have been excluded.

. DOI: 10.1107/S2056989015018885/hb7489fig2.tif
Unit cell packing of the title compound showing inter­molecular C—H⋯O inter­actions with dotted lines. H-atoms not involved in hydrogen bonding have been excluded.

**Fig. 3.**
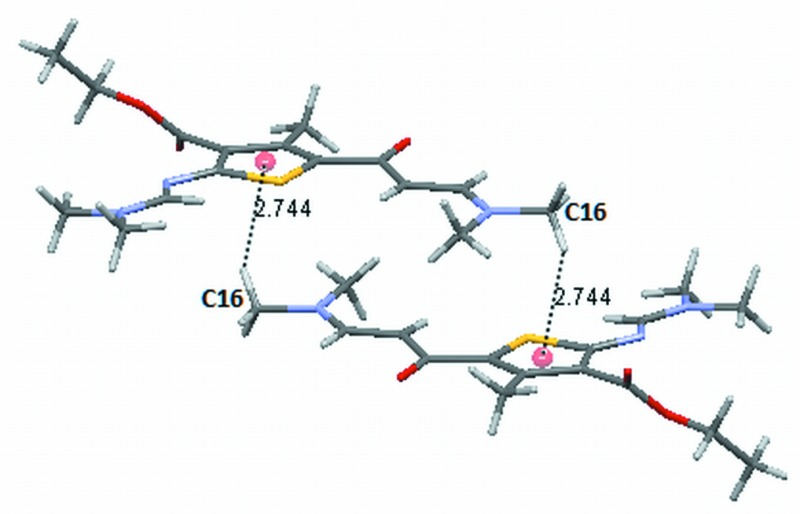
Unit-cell packing depicting the intermolecular C—H···π interactions with dotted lines.

. DOI: 10.1107/S2056989015018885/hb7489fig3.tif
Unit-cell packing depicting the inter­molecular C—H⋯π inter­actions with dotted lines.

CCDC reference: 1430038

Additional supporting information:  crystallographic information; 3D view; checkCIF report

## Figures and Tables

**Table 1:** Hydrogen-bond geometry (Å, °) *Cg* is the centroid of the C2/C3/C4/C5/S1 ring.

*D*—H⋯*A*	*D*—H	H⋯*A*	*D*⋯*A*	*D*—H⋯*A*
C11—H11*B*⋯O1	0.98	2.31	3.054 (1)	132
C16—H16*C*⋯O3^i^	0.98	2.35	3.310 (2)	168
C16—H16*B*⋯*Cg* ^ii^	0.98	2.74	3.566 (2)	142

Symmetry codes: (i) 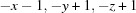; (ii) 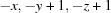.

## supplementary crystallographic information

### S1. Comment

Sulfur containing heterocycles are seen as the center of activity due to their widespread use in several important medicinal compounds. However, it is seen that the success of thiophene as an important moiety of medicinal agents led to the introduction of new therapeutic drugs. Substituted thiophene derivatives are well known for their chemotherapeutic applications (Mabkhot *et al.*, 2013; Mishra *et al.*, 2011). Many thiophene based heterocyclic compounds have shown versatile pharmacological activities such as antimicrobial, antiamoebic, antiparasitic, anticancer, diabetes mellitus, analgesic, antidepressant and antiallergic. In addition, the cholesterol inhibition activity and as antagonist against many hormones releasing receptors has also been reported (Rizwan *et al.*, 2014). 2-Aminothiophenes attract special attention because of their applications in pharmaceuticals, agriculture, pesticides and dyes (Sabnis *et al.*, 1999). The most convergent and well established classical approach for the preparation of 2-aminothiophenes is Gewald's method (Gewald *et al.*, 1966). Herein, we report the structure of the title compound (I).

### S2. Experimental

A mixture of ethyl 5-acetyl-2-amino-4-methyl-thiophene-3-carboxylate (10 mmol) and DMF—DMA (5 ml) was heated under reflux for 2 h. To this add ethanol and kept in room temperature to give a solid product (title compound) that was collected by filtration. The compound was recrystallized by slow evaporation from ethanol, yielding colourless blocks.

### S3. Refinement

The H atoms were placed at calculated positions in the riding-model approximation with C—H = 0.96° A, 0.97 ° A and 0.93 ° A for methyl, methylene and methyne H-atoms respectively, with *U*_iso_(H) = 1.5*U*_eq_(C) for methyl H atoms and *U*_iso_(H) = 1.2Ueq(C) for other hydrogen atoms.

### Crystal data

**Table:**

C_16_H_23_N_3_O_3_S	*Z* = 2
*M**_r_* = 337.43	*F*(000) = 360
Triclinic, *P*1	*D*_x_ = 1.320 Mg m^−^^3^
Hall symbol: -P 1	Mo *K*α radiation, λ = 0.71073 Å
*a* = 7.6954 (5) Å	Cell parameters from 2991 reflections
*b* = 8.1799 (5) Å	θ = 2.5–25.0°
*c* = 13.9626 (9) Å	µ = 0.21 mm^−^^1^
α = 95.928 (2)°	*T* = 100 K
β = 103.685 (2)°	Block, colorless
γ = 90.137 (2)°	0.17 × 0.16 × 0.15 mm
*V* = 849.07 (9) Å^3^	

### Data collection

**Table:**

Bruker SMART APEX CCD diffractometer	2991 independent reflections
Radiation source: fine-focus sealed tube	2646 reflections with *I* > 2σ(*I*)
Graphite monochromator	*R*_int_ = 0.016
ω scans	θ_max_ = 25.0°, θ_min_ = 2.5°
Absorption correction: multi-scan (*SADABS*; Bruker, 1998)	*h* = −9→8
*T*_min_ = 0.963, *T*_max_ = 0.967	*k* = −9→9
5876 measured reflections	*l* = −11→16

### Refinement

**Table:**

Refinement on *F*^2^	Primary atom site location: structure-invariant direct methods
Least-squares matrix: full	Secondary atom site location: difference Fourier map
*R*[*F*^2^ > 2σ(*F*^2^)] = 0.032	Hydrogen site location: inferred from neighbouring sites
*wR*(*F*^2^) = 0.089	H-atom parameters constrained
*S* = 1.02	*w* = 1/[σ^2^(*F*_o_^2^) + (0.0528*P*)^2^ + 0.3093*P*] where *P* = (*F*_o_^2^ + 2*F*_c_^2^)/3
2991 reflections	(Δ/σ)_max_ < 0.001
214 parameters	Δρ_max_ = 0.27 e Å^−^^3^
0 restraints	Δρ_min_ = −0.19 e Å^−^^3^

### Special details

**Table:**

Geometry. All s.u.'s (except the s.u. in the dihedral angle between two l.s. planes) are estimated using the full covariance matrix. The cell s.u.'s are taken into account individually in the estimation of s.u.'s in distances, angles and torsion angles; correlations between s.u.'s in cell parameters are only used when they are defined by crystal symmetry. An approximate (isotropic) treatment of cell s.u.'s is used for estimating s.u.'s involving l.s. planes.
Refinement. Refinement of *F*^2^ against ALL reflections. The weighted *R*-factor *wR* and goodness of fit *S* are based on *F*^2^, conventional *R*-factors *R* are based on *F*, with *F* set to zero for negative *F*^2^. The threshold expression of *F*^2^ > 2σ(*F*^2^) is used only for calculating *R*-factors(gt) *etc*. and is not relevant to the choice of reflections for refinement. *R*-factors based on *F*^2^ are statistically about twice as large as those based on *F*, and *R*- factors based on ALL data will be even larger.

### Fractional atomic coordinates and isotropic or equivalent isotropic displacement parameters (Å^2^)

**Table:**

	*x*	*y*	*z*	*U*_iso_*/*U*_eq_	
S1	0.02778 (5)	0.86621 (4)	0.39020 (3)	0.01529 (13)	
O1	−0.26349 (16)	0.82247 (14)	0.03518 (8)	0.0269 (3)	
O2	−0.15686 (14)	1.07646 (13)	0.09662 (7)	0.0181 (3)	
O3	−0.41534 (15)	0.62783 (15)	0.39383 (8)	0.0255 (3)	
N1	0.15977 (16)	1.01180 (15)	0.25146 (9)	0.0150 (3)	
N2	0.44236 (16)	1.13376 (16)	0.31336 (9)	0.0172 (3)	
N3	−0.10460 (16)	0.56379 (15)	0.67393 (9)	0.0162 (3)	
C1	0.2971 (2)	1.05575 (18)	0.32371 (11)	0.0157 (3)	
H1	0.2938	1.0306	0.3882	0.019*	
C2	0.0200 (2)	0.93094 (17)	0.27440 (11)	0.0140 (3)	
C3	−0.14156 (19)	0.88099 (18)	0.20962 (11)	0.0142 (3)	
C4	−0.2563 (2)	0.78787 (18)	0.25248 (11)	0.0147 (3)	
C5	−0.18389 (19)	0.77234 (18)	0.35108 (11)	0.0147 (3)	
C6	0.4627 (2)	1.1762 (2)	0.21829 (12)	0.0213 (4)	
H6A	0.5572	1.1107	0.1983	0.032*	
H6B	0.4950	1.2934	0.2238	0.032*	
H6C	0.3497	1.1533	0.1685	0.032*	
C7	0.5897 (2)	1.1801 (2)	0.39906 (12)	0.0242 (4)	
H7A	0.5567	1.1532	0.4593	0.036*	
H7B	0.6153	1.2986	0.4042	0.036*	
H7C	0.6963	1.1198	0.3913	0.036*	
C8	−0.19266 (19)	0.91884 (19)	0.10487 (11)	0.0164 (3)	
C9	−0.2086 (2)	1.1249 (2)	−0.00331 (11)	0.0221 (4)	
H9A	−0.3396	1.1106	−0.0295	0.027*	
H9B	−0.1485	1.0563	−0.0478	0.027*	
C10	−0.1529 (3)	1.3026 (2)	0.00224 (13)	0.0306 (4)	
H10A	−0.2159	1.3696	0.0449	0.046*	
H10B	−0.1830	1.3384	−0.0644	0.046*	
H10C	−0.0236	1.3155	0.0299	0.046*	
C11	−0.4355 (2)	0.7165 (2)	0.19573 (12)	0.0197 (3)	
H11A	−0.4507	0.6055	0.2135	0.030*	
H11B	−0.4428	0.7115	0.1245	0.030*	
H11C	−0.5302	0.7860	0.2122	0.030*	
C12	−0.2594 (2)	0.68572 (18)	0.42080 (11)	0.0158 (3)	
C13	−0.1447 (2)	0.67102 (18)	0.51655 (11)	0.0160 (3)	
H13	−0.0280	0.7203	0.5334	0.019*	
C14	−0.2011 (2)	0.58678 (18)	0.58402 (11)	0.0154 (3)	
H14	−0.3192	0.5408	0.5650	0.018*	
C15	0.0778 (2)	0.62890 (19)	0.70973 (11)	0.0186 (3)	
H15A	0.1460	0.5980	0.6599	0.028*	
H15B	0.1339	0.5836	0.7716	0.028*	
H15C	0.0765	0.7490	0.7218	0.028*	
C16	−0.1802 (2)	0.48165 (19)	0.74298 (11)	0.0195 (3)	
H16A	−0.1883	0.5607	0.7994	0.029*	
H16B	−0.1032	0.3918	0.7667	0.029*	
H16C	−0.3000	0.4372	0.7094	0.029*	

### Atomic displacement parameters (Å^2^)

**Table:**

	*U*^11^	*U*^22^	*U*^33^	*U*^12^	*U*^13^	*U*^23^
S1	0.0142 (2)	0.0190 (2)	0.0122 (2)	−0.00330 (14)	0.00129 (14)	0.00392 (14)
O1	0.0338 (7)	0.0294 (7)	0.0144 (6)	−0.0129 (5)	0.0000 (5)	0.0008 (5)
O2	0.0211 (6)	0.0210 (6)	0.0116 (5)	−0.0003 (4)	0.0010 (4)	0.0065 (4)
O3	0.0176 (6)	0.0392 (7)	0.0192 (6)	−0.0092 (5)	0.0004 (5)	0.0112 (5)
N1	0.0134 (6)	0.0177 (7)	0.0143 (6)	−0.0007 (5)	0.0030 (5)	0.0040 (5)
N2	0.0133 (6)	0.0213 (7)	0.0166 (7)	−0.0028 (5)	0.0021 (5)	0.0031 (5)
N3	0.0149 (7)	0.0177 (7)	0.0160 (7)	−0.0015 (5)	0.0027 (5)	0.0044 (5)
C1	0.0156 (8)	0.0169 (8)	0.0156 (8)	0.0006 (6)	0.0046 (6)	0.0037 (6)
C2	0.0157 (7)	0.0131 (7)	0.0136 (7)	0.0021 (6)	0.0038 (6)	0.0024 (6)
C3	0.0143 (7)	0.0140 (7)	0.0141 (8)	0.0007 (6)	0.0027 (6)	0.0025 (6)
C4	0.0149 (7)	0.0133 (7)	0.0161 (8)	0.0012 (6)	0.0033 (6)	0.0031 (6)
C5	0.0129 (7)	0.0144 (7)	0.0160 (8)	0.0000 (6)	0.0015 (6)	0.0018 (6)
C6	0.0198 (8)	0.0243 (8)	0.0216 (8)	−0.0025 (7)	0.0073 (7)	0.0051 (7)
C7	0.0169 (8)	0.0336 (9)	0.0199 (9)	−0.0066 (7)	0.0000 (7)	0.0031 (7)
C8	0.0121 (7)	0.0214 (8)	0.0163 (8)	0.0001 (6)	0.0038 (6)	0.0042 (6)
C9	0.0219 (8)	0.0314 (9)	0.0119 (8)	−0.0030 (7)	−0.0011 (6)	0.0094 (7)
C10	0.0370 (10)	0.0328 (10)	0.0211 (9)	−0.0046 (8)	0.0008 (8)	0.0128 (7)
C11	0.0158 (8)	0.0253 (8)	0.0177 (8)	−0.0034 (6)	0.0009 (6)	0.0076 (6)
C12	0.0161 (8)	0.0147 (8)	0.0172 (8)	0.0002 (6)	0.0050 (6)	0.0022 (6)
C13	0.0145 (8)	0.0169 (8)	0.0163 (8)	−0.0027 (6)	0.0024 (6)	0.0033 (6)
C14	0.0151 (7)	0.0145 (7)	0.0158 (8)	0.0001 (6)	0.0024 (6)	0.0013 (6)
C15	0.0164 (8)	0.0215 (8)	0.0164 (8)	−0.0016 (6)	−0.0001 (6)	0.0043 (6)
C16	0.0206 (8)	0.0229 (8)	0.0163 (8)	−0.0007 (6)	0.0049 (6)	0.0070 (6)

### Geometric parameters (Å, º)

**Table:**

S1—C5	1.7401 (15)	C6—H6C	0.9800
S1—C2	1.7405 (15)	C7—H7A	0.9800
O1—C8	1.2058 (19)	C7—H7B	0.9800
O2—C8	1.3399 (19)	C7—H7C	0.9800
O2—C9	1.4541 (18)	C9—C10	1.503 (2)
O3—C12	1.2458 (19)	C9—H9A	0.9900
N1—C1	1.2973 (19)	C9—H9B	0.9900
N1—C2	1.3785 (19)	C10—H10A	0.9800
N2—C1	1.330 (2)	C10—H10B	0.9800
N2—C6	1.449 (2)	C10—H10C	0.9800
N2—C7	1.4574 (19)	C11—H11A	0.9800
N3—C14	1.3308 (19)	C11—H11B	0.9800
N3—C15	1.4538 (19)	C11—H11C	0.9800
N3—C16	1.4547 (19)	C12—C13	1.434 (2)
C1—H1	0.9500	C13—C14	1.370 (2)
C2—C3	1.386 (2)	C13—H13	0.9500
C3—C4	1.435 (2)	C14—H14	0.9500
C3—C8	1.487 (2)	C15—H15A	0.9800
C4—C5	1.377 (2)	C15—H15B	0.9800
C4—C11	1.502 (2)	C15—H15C	0.9800
C5—C12	1.482 (2)	C16—H16A	0.9800
C6—H6A	0.9800	C16—H16B	0.9800
C6—H6B	0.9800	C16—H16C	0.9800
			
C5—S1—C2	92.90 (7)	O2—C9—C10	107.39 (13)
C8—O2—C9	115.32 (12)	O2—C9—H9A	110.2
C1—N1—C2	117.13 (13)	C10—C9—H9A	110.2
C1—N2—C6	122.52 (13)	O2—C9—H9B	110.2
C1—N2—C7	120.47 (13)	C10—C9—H9B	110.2
C6—N2—C7	117.01 (13)	H9A—C9—H9B	108.5
C14—N3—C15	121.29 (12)	C9—C10—H10A	109.5
C14—N3—C16	121.63 (13)	C9—C10—H10B	109.5
C15—N3—C16	116.97 (12)	H10A—C10—H10B	109.5
N1—C1—N2	124.23 (14)	C9—C10—H10C	109.5
N1—C1—H1	117.9	H10A—C10—H10C	109.5
N2—C1—H1	117.9	H10B—C10—H10C	109.5
N1—C2—C3	126.29 (13)	C4—C11—H11A	109.5
N1—C2—S1	123.88 (11)	C4—C11—H11B	109.5
C3—C2—S1	109.75 (11)	H11A—C11—H11B	109.5
C2—C3—C4	113.91 (13)	C4—C11—H11C	109.5
C2—C3—C8	123.54 (13)	H11A—C11—H11C	109.5
C4—C3—C8	122.55 (13)	H11B—C11—H11C	109.5
C5—C4—C3	112.46 (13)	O3—C12—C13	123.52 (14)
C5—C4—C11	124.08 (14)	O3—C12—C5	119.47 (13)
C3—C4—C11	123.46 (13)	C13—C12—C5	117.00 (13)
C4—C5—C12	128.92 (14)	C14—C13—C12	120.85 (14)
C4—C5—S1	110.95 (11)	C14—C13—H13	119.6
C12—C5—S1	120.09 (11)	C12—C13—H13	119.6
N2—C6—H6A	109.5	N3—C14—C13	125.64 (14)
N2—C6—H6B	109.5	N3—C14—H14	117.2
H6A—C6—H6B	109.5	C13—C14—H14	117.2
N2—C6—H6C	109.5	N3—C15—H15A	109.5
H6A—C6—H6C	109.5	N3—C15—H15B	109.5
H6B—C6—H6C	109.5	H15A—C15—H15B	109.5
N2—C7—H7A	109.5	N3—C15—H15C	109.5
N2—C7—H7B	109.5	H15A—C15—H15C	109.5
H7A—C7—H7B	109.5	H15B—C15—H15C	109.5
N2—C7—H7C	109.5	N3—C16—H16A	109.5
H7A—C7—H7C	109.5	N3—C16—H16B	109.5
H7B—C7—H7C	109.5	H16A—C16—H16B	109.5
O1—C8—O2	123.15 (14)	N3—C16—H16C	109.5
O1—C8—C3	124.90 (14)	H16A—C16—H16C	109.5
O2—C8—C3	111.90 (13)	H16B—C16—H16C	109.5
			
C2—N1—C1—N2	179.64 (14)	C2—S1—C5—C4	0.96 (12)
C6—N2—C1—N1	−0.8 (2)	C2—S1—C5—C12	179.02 (12)
C7—N2—C1—N1	179.29 (14)	C9—O2—C8—O1	−0.3 (2)
C1—N1—C2—C3	176.62 (14)	C9—O2—C8—C3	−178.01 (12)
C1—N1—C2—S1	−7.12 (19)	C2—C3—C8—O1	136.70 (17)
C5—S1—C2—N1	−176.53 (13)	C4—C3—C8—O1	−42.9 (2)
C5—S1—C2—C3	0.27 (11)	C2—C3—C8—O2	−45.7 (2)
N1—C2—C3—C4	175.30 (13)	C4—C3—C8—O2	134.66 (14)
S1—C2—C3—C4	−1.41 (16)	C8—O2—C9—C10	−178.30 (13)
N1—C2—C3—C8	−4.4 (2)	C4—C5—C12—O3	−6.8 (2)
S1—C2—C3—C8	178.91 (11)	S1—C5—C12—O3	175.57 (12)
C2—C3—C4—C5	2.19 (19)	C4—C5—C12—C13	172.14 (15)
C8—C3—C4—C5	−178.13 (13)	S1—C5—C12—C13	−5.54 (19)
C2—C3—C4—C11	−178.12 (14)	O3—C12—C13—C14	1.6 (2)
C8—C3—C4—C11	1.6 (2)	C5—C12—C13—C14	−177.21 (13)
C3—C4—C5—C12	−179.75 (14)	C15—N3—C14—C13	−0.2 (2)
C11—C4—C5—C12	0.6 (3)	C16—N3—C14—C13	175.89 (14)
C3—C4—C5—S1	−1.90 (16)	C12—C13—C14—N3	178.91 (14)
C11—C4—C5—S1	178.41 (12)		

### Hydrogen-bond geometry (Å, º)

Cg is the centroid of the C2/C3/C4/C5/S1 ring.

**Table:**

*D*—H···*A*	*D*—H	H···*A*	*D*···*A*	*D*—H···*A*
C11—H11*B*···O1	0.98	2.31	3.054 (1)	132
C16—H16*C*···O3^i^	0.98	2.35	3.310 (2)	168
C16—H16*B*···*Cg*^ii^	0.98	2.74	3.566 (2)	142

Symmetry codes: (i) −*x*−1, −*y*+1, −*z*+1; (ii) −*x*, −*y*+1, −*z*+1.

## References

[bb2] Bruker. (1998). *SMART*, *SAINT-Plus* and *SADABS.* Bruker Axs Inc., Madison, Wisconsin, USA.

[bb3] Farrugia, L. J. (2012). *J. Appl. Cryst.* **45**, 849–854.

[bb4] Gewald, K., Schinke, E. & Böttcher, H. (1966). *Chem. Ber.* **99**, 94–100.

[bb5] Mabkhot, Y. N., Barakat, A., Al-Majid, A. & Choudhary, M. I. (2013). *Int. J. Mol. Sci.* **14**, 5712–5722. 10.3390/ijms14035712 PMC3634413 23481634

[bb6] Mishra, R., Jha, K. K., Kumar, S. & Tomer, S. (2011). *Pharma Chem.* **3**, 38–54.

[bb7] Rizwan, K., Zubair, M., Rasool, N., Ali, S., Zahoor, A. F., Rana, U. A., Khan, S. U., Shahid, M., Zia-Ul-Haq, M. & Jaafar, H. Z. (2014). *Chem. Cent. J.* **8**, 74. 10.1186/s13065-014-0074-z PMC4326645 25685184

[bb8] Sabnis, R. W., Rangnekar, D. W. & Sonawane, N. D. (1999). *J. Heterocycl. Chem.* **36**, 333–345.

[bb9] Sheldrick, G. M. (2008). *Acta Cryst.* A**64**, 112–122. 10.1107/S0108767307043930 18156677

[bb10] Watkin, D. J., Prout, C. K. & Pearce, L. J. (1996). *CAMERON.* Chemical Crystallography Laboratory, University of Oxford, England.

