# Supplementary figures and images for: Crystal structure of ethyl 5-[3-(di­methyl­amino)­acrylo­yl]-2-{[(di­methyl­amino)­methyl­idene]­amino}-4-methylthio­phene-3-carb­oxy­late
Source: Acta Crystallogr E Crystallogr Commun. 2015 Nov 4;71(Pt 12):o908–9. doi: 10.1107/S2056989015018885 (PMC4719874; doi:10.1107/S2056989015018885)

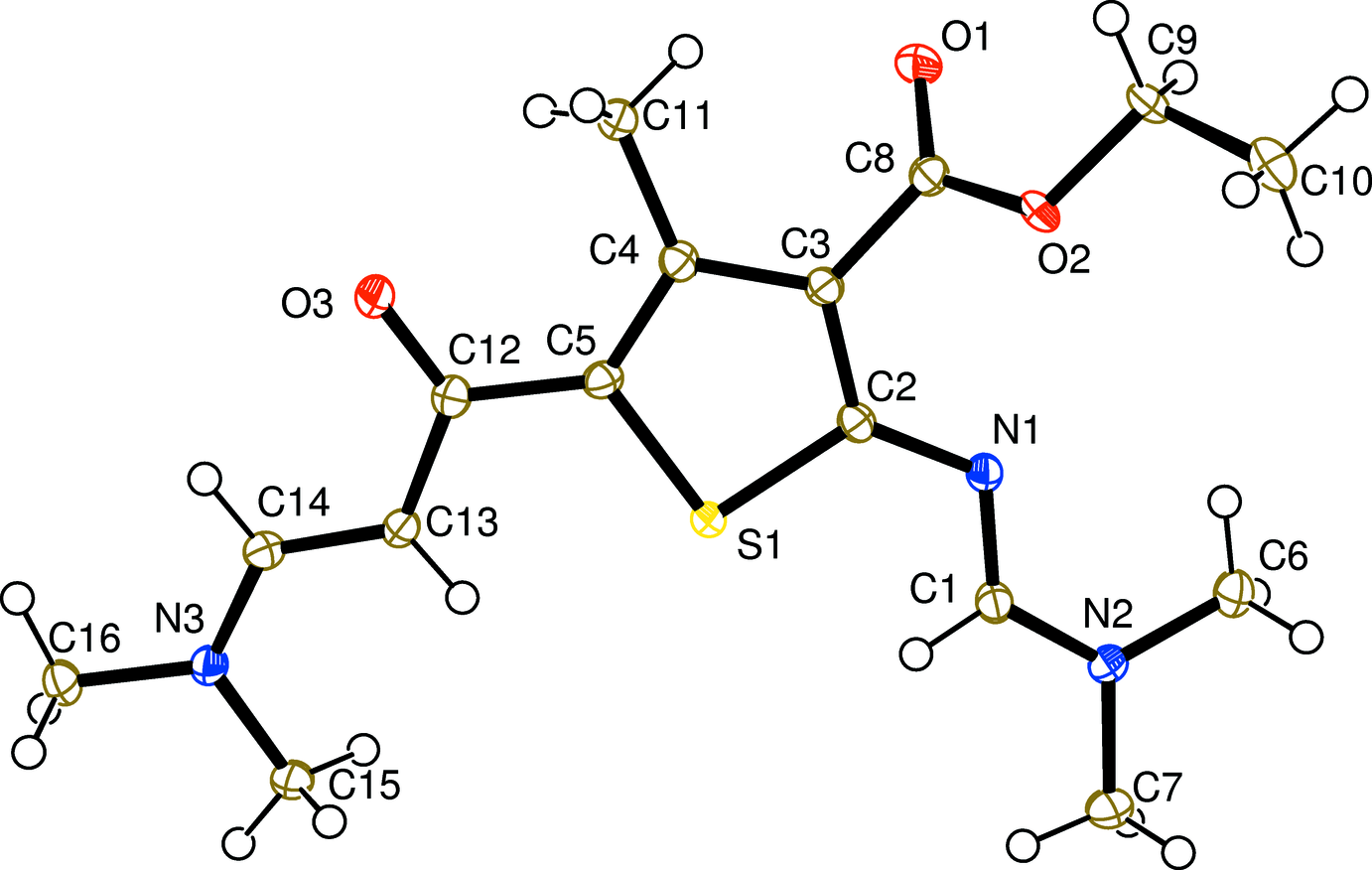

Supplement: Supplementary file 4 [file e-71-0o908-fig1.tif]

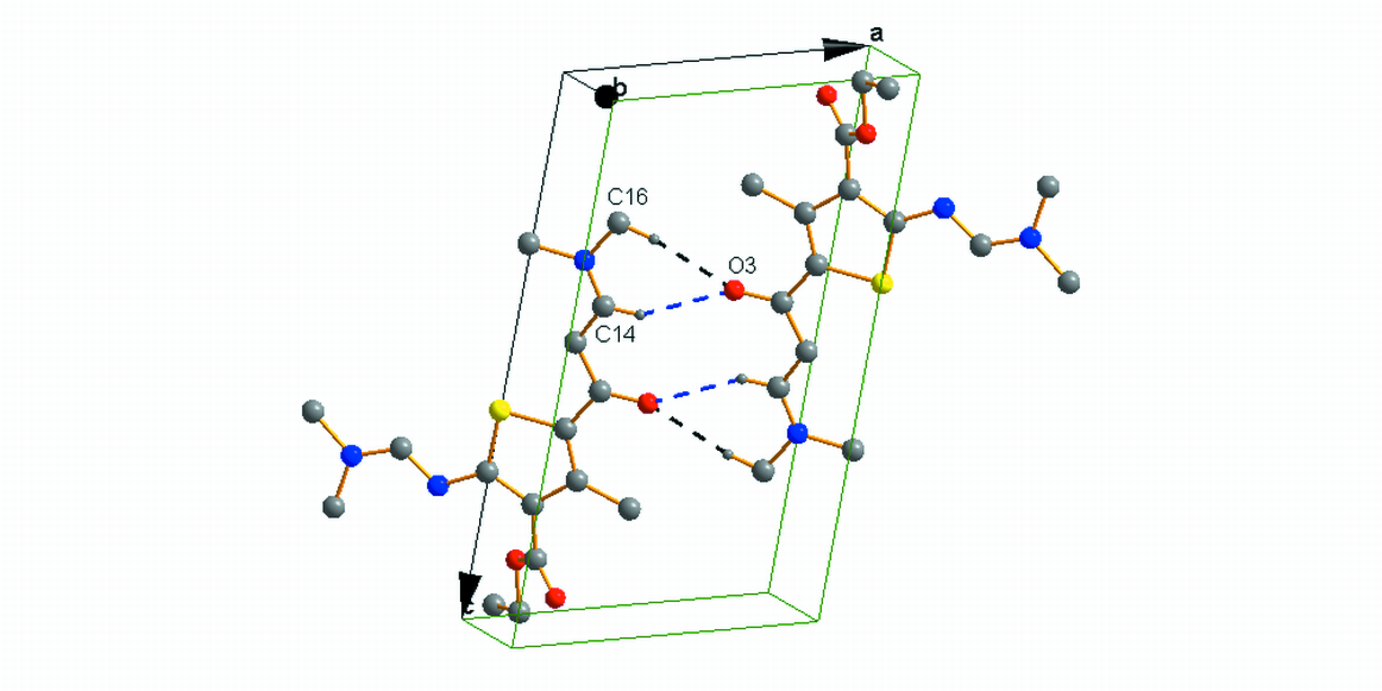

Supplement: Supplementary file 5 [file e-71-0o908-fig2.tif]

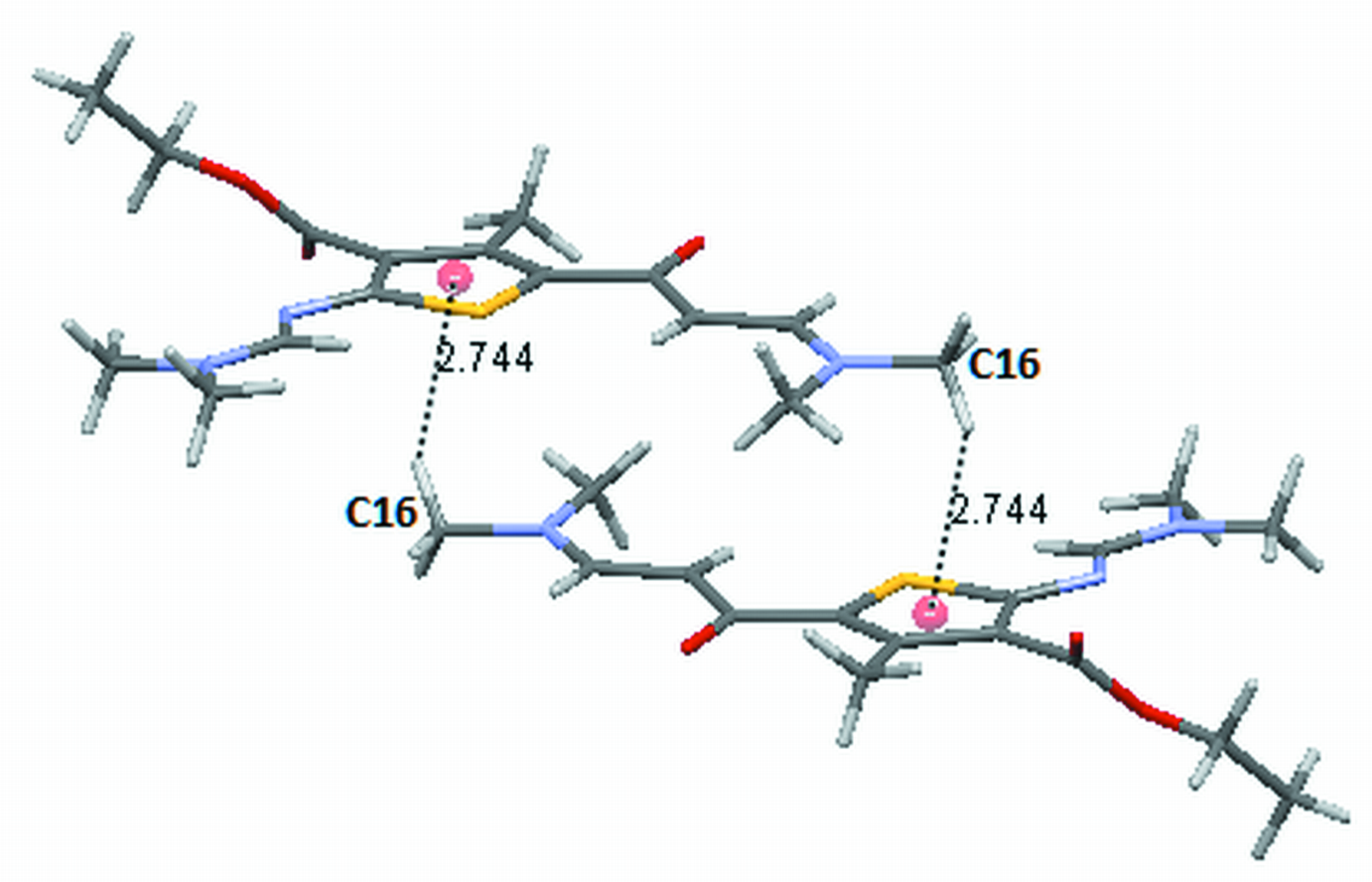

Supplement: Supplementary file 6 [file e-71-0o908-fig3.tif]
